# The signer and the sign: Cortical correlates of person identity and language processing from point-light displays

**DOI:** 10.1016/j.neuropsychologia.2011.06.029

**Published:** 2011-09

**Authors:** Ruth Campbell, Cheryl M. Capek, Karine Gazarian, Mairéad MacSweeney, Bencie Woll, Anthony S. David, Philip K. McGuire, Michael J. Brammer

**Affiliations:** a ESRC Deafness, Cognition and Language Research Centre (DCAL), Division of Psychology and Language Sciences, University College London, 49 Gordon Square, London WC1H 0PD, UK; b School of Psychological Sciences, University of Manchester, Zochonis Building, Manchester M13 9PL, UK; c Wellcome Trust Centre for Neuroimaging, University College London, 12 Queen Square, London WC1N 3BG, UK; d Institute of Cognitive Neuroscience, University College London, 17 Queen Square, London WC1N 3AR, UK; e Institute of Psychiatry, Kings College London, De Crespigny Park, London SE5 8AF, UK

**Keywords:** Sign language, Biological motion perception, fMRI, Point-light

## Abstract

In this study, the first to explore the cortical correlates of signed language (SL) processing under point-light display conditions, the observer identified either a signer or a lexical sign from a display in which different signers were seen producing a number of different individual signs. Many of the regions activated by point-light under these conditions replicated those previously reported for full-image displays, including regions within the inferior temporal cortex that are specialised for face and body-part identification, although such body parts were invisible in the display. Right frontal regions were also recruited – a pattern not usually seen in full-image SL processing. This activation may reflect the recruitment of information about person identity from the reduced display. A direct comparison of identify-signer and identify-sign conditions showed these tasks relied to a different extent on the posterior inferior regions. Signer identification elicited greater activation than sign identification in (bilateral) inferior temporal gyri (BA 37/19), fusiform gyri (BA 37), middle and posterior portions of the middle temporal gyri (BAs 37 and 19), and superior temporal gyri (BA 22 and 42). Right inferior frontal cortex was a further focus of differential activation (signer > sign).

These findings suggest that the neural systems supporting point-light displays for the processing of SL rely on a cortical network including areas of the inferior temporal cortex specialized for face and body identification. While this might be predicted from other studies of whole body point-light actions (Vaina, Solomon, Chowdhury, Sinha, & Belliveau, 2001) it is not predicted from the perspective of spoken language processing, where voice characteristics and speech content recruit distinct cortical regions (Stevens, 2004) in addition to a common network. In this respect, our findings contrast with studies of voice/speech recognition (Von Kriegstein, Kleinschmidt, Sterzer, & Giraud, 2005). Inferior temporal regions associated with the visual recognition of a person appear to be required during SL processing, for both carrier and content information.

## Highlights

► First cortical imaging study of point-light signed language (SL). ► Distinguishes carrier and content in SL. ► Describes cortical circuitry for both carrier and content in SL.

## 1 Introduction

Patterns of biological motion are among the most salient of all visual signals, and are readily perceived from sparse displays. Johansson (1973, 1976) showed that lights mounted on the rigid joints of a human walker or dancer were identified as human motion patterns within half a second of the display movement onset, despite a complete lack of pictorial detail. When seen as stilled frames such displays are uninterpretable, yet once in motion the action becomes comprehensible. Point-light displays of biological motion can support the perception of a variety of action patterns such as walking, running and dancing (Dittrich, 1993), as well as idiosyncratic characteristics of the actor, which can be gleaned from those movements. These include not only gender and mood (e.g. Cutting, 1978; Dittrich, Troscianko, Lea, & Morgan, 1996; Pollick, Lestou, Ryu, & Cho, 2002) but also identifying a familiar person from their gait or facial actions (Cutting & Kozlowski, 1977; Hill & Johnston, 2001; Loula, Prasad, Harber, & Shiffrar, 2005; Rosenblum, Smith, Nichols, Hale, & Lee, 2006; Rosenblum, Niehus, & Smith, 2007). Biological motion can also deliver linguistic content. Displays comprising head and shoulder markers and illumination of points on the rigid joints of arms, head and fingers, are ‘readable’ as sign language (SL) utterances for skilled signers (Poizner, Bellugi, & Lutes-Driscoll, 1981; Tartter & Fischer, 1982; Tartter & Knowlton, 1981). Thus, point-light displays can be used as a tool for removing direct, image based information of the person and to explore the extent to which neural systems recruited for SL processing are affected when only a minimal amount of pictorial information is available.

In signed conversations the source of the message is always visible. Thus, studies of natural SL processing may reflect not only linguistic content, but also aspects of the signer's identity, which may be processed along with the linguistic message. A useful comparison here is with auditory speech. The talker's identity, as well as the content of the spoken message, can often be accurately perceived from the voice alone (Allen et al., 2005; Belin, Fecteau, & Bedard, 2004; Fu et al., 2006). Neuroimaging studies of speech processing reveal distinctive circuits for identification of the content of the vocal signal and for its carrier (e.g. Von Kriegstein & Giraud, 2004). In particular right anterior superior temporal regions are more sensitive to voice-identity than content-identity (Von Kriegstein, Eger, Kleinschmidt, & Giraud, 2003). Other clearly visual, non-auditory regions, including inferior temporal and fusiform gyri which are sensitive to face identification, can also be activated by known voices. Critically, recruitment of these face and body processing regions is only elicited when the task is identification of the talker and when the voice is that of a known person or a learned face/voice association (Von Kriegstein & Giraud, 2004; Von Kriegstein et al., 2005, 2008). Thus, the identification of a familiar voice can activate visual circuitry relevant to that decision. By contrast, identifying the linguistic *content* of a spoken utterance does not activate these regions – even when spoken by a familiar voice (von Kriegstein et al., 2004; Von Kriegstein et al., 2008).

Taking a similar approach to signed language, here we address whether temporal regions implicated in identity processing for voice (right anterior temporal; bilateral fusiform and lingual gyri) may be engaged especially during identification of the signer compared with identification of the sign, when the person information available has been reduced by using point-light presentation of signs.

More generally, point-light displays of SL allow us to explore the extent to which regions implicated in natural SL processing are also engaged when the pictorial correlates of the displays are reduced to a minimum, leaving just the kinematics of the sign utterance to ‘carry the message’. The cortical correlates of whole body biological motion delivered through point-light displays have been extensively explored (Beauchamp, Lee, Haxby, & Martin, 2003; Bonda, Petrides, Ostry, & Evans, 1996; Grèzes et al., 2001; Grossman & Blake, 2002; Grossman et al., 2000; Oram & Perrett, 1994; Saygin, 2007; Vaina et al., 2001). Several regions are engaged when a point-light body in motion is perceived. Most reliably, perceiving point-light body motion activates posterior parts of the superior temporal sulcus (STS-p). The specificity of activation in this region is suggested by contrasts with other high-level motion displays that cannot be interpreted as biological actions. STS-p responds similarly to point-light displays and pictorial videos that contain equivalent biological motion (Beauchamp et al., 2003). STS-p is a projection site from occipito-temporal regions specialised for the processing of visual motion more generally, that is, it can be considered to be a projection site for the dorsal visual system. These occipito-temporal regions, especially areas V5 (MT) and dorsal V3, are necessarily involved in point-light biological motion perception (Grossman et al., 2000; Vaina et al., 2001). There are also reports of activation in superior parietal and parieto-frontal circuits (Bonda et al., 1996; Saygin, Wilson, Hagler, Bates, & Sereno, 2004), suggestive of a circuit whose functional basis includes mirroring of seen complex actions (Molnar-Szakacs, Kaplan, Greenfield, & Iacoboni, 2006; Ulloa & Pineda, 2007). STS-p is not just a crucial region for natural (i.e. audiovisual, face-to-face) language (Campbell, 2008) and biological motion processing. While its motion processing function reflects its input from the dorsal visual system in earlier visual processing areas (V5, dorsal V3), it is also a projection site for neural processing from extrastriate occipital and inferior temporal regions forming part of the ventral visual processing system, including lingual and fusiform gyri. These regions have been shown to be activated by point-light illuminations of moving bodies and body parts in several studies (e.g. Beauchamp et al., 2003; Grossman & Blake, 2002; Peelen, Wiggett, & Downing, 2006; Santi, Servos, Vatikiotis-Bateson, Kuratate, & Munhall, 2003), reflecting their involvement in the processing of pictorial information relevant to person perception, despite the absence of pictorial detail in the display (e.g. Beauchamp et al., 2003; Downing et al., 2007).

Natural displays of SL engage the core language network in the left perisylvian cortex (see MacSweeney, Capek, Campbell, & Woll, 2008 for review). In addition, as would be expected, SL processing engages many of the regions activated by the perception of biological motion, including V5 and STS-p (e.g. Capek et al., 2008; Corina & Knapp, 2006; Corina et al., 2007; Lambertz, Gizewski, de Greiff, & Forsting, 2005; MacSweeney et al., 2002; Neville et al., 1998; Newman, Bavelier, Corina, Jezzard, & Neville, 2002; Petitto et al., 2000; Sakai, Tatsuno, Suzuki, Kimura, & Ichida, 2005). Activation here is relatively enhanced in linguistic contrasts, e.g. signs > nonsense-signs (Neville et al., 1998; Newman et al., 2002; Petitto et al., 2000); and signs > non-linguistic actions (Corina et al., 2007; MacSweeney et al., 2004). Greater activation is found in superior temporal regions for signed sentences than for lists of single signs (MacSweeney et al., 2006). Similarly, inferior temporal visual regions (posterior inferior temporal and fusiform gyri) which are implicated in processing visual form, are also important in linguistic processing in SL (e.g. Capek et al., 2008; Waters et al., 2007). All this suggests that point-light displays should activate many parts of the cortical circuits already identified for full pictorial displays of SL.

However, the majority of previous studies of point-light biological motion processing have presented whole bodies in movement (e.g. Beauchamp et al., 2003; Grossman & Blake, 2002; Peelen et al., 2006; Santi et al., 2003). In contrast, in the present study the head and torso are relatively still, and just the hands and arms move to produce distinctive signed forms. We predicted that regions involved in biological motion and SL processing, especially STS-p, would be activated under these conditions, whether the task was to identify the signer or the sign. However, the extent to which other regions which project to STS-p, for instance parts of the lingual and fusiform gyri in the inferior temporal cortex which respond preferentially to faces, animals, bodies, – is unknown.

To summarize, in this study we explored the cortical correlates for the perception of single British Sign Language (BSL) signs presented by different signers as point-light displays. Previous studies both of point-light biological movement, and of the processing of SL under natural display conditions suggest cortical circuitry involving STS-p and several inferior temporal and temporo-occipital regions, and this study investigated the extent to which these regions were activated when observing SL point-light displays of hand and arm actions (only). An important focus of this study was to establish the extent to which distinctive activation occurred when the viewer identified the sign and when he identified the signer. By analogy with studies of voice processing (Von Kriegstein et al., 2005), we predicted that inferior temporo-occipital regions implicated in face and body processing would be engaged during identification of the signer to a greater extent than during identification of the sign.

## 2 Methods

In this study, respondents were asked, in one experimental run, to identify a particular signer, and in another, to identify a given sign. The material was the same for both tasks, but the participant was directed to attend to one or other aspect of the display depending on the experimental condition (see Von Kriegstein & Giraud, 2004 for similar rationale). For details of experimental procedure see below.

### 2.1 Participants

Twelve (five female; mean age: 25.2; age range: 18–30) right-handed volunteers were tested. Volunteers were congenitally, severely or profoundly deaf (81 dB mean loss or greater in the better ear over four octaves, spanning 500–4000 Hz). Across the group, the mean hearing loss in the better ear was 105 dB. All but one participant had some experience with hearing aids. Eight only used hearing aids at school: four continued to wear them as adults. The participants were native signers, having acquired BSL from their deaf parents. None of the participants had any known neurological or behavioural abnormalities and they performed above average on NVIQ (centile range = 75–99.9), as measured by the Block Design subtest of the WAIS-R.

All volunteers gave written informed consent to participate in the study, which was approved by the Institute of Psychiatry/South London and Maudsley NHS Trust Research Ethics Committee.

### 2.2 Stimuli

**Fig. 1:**
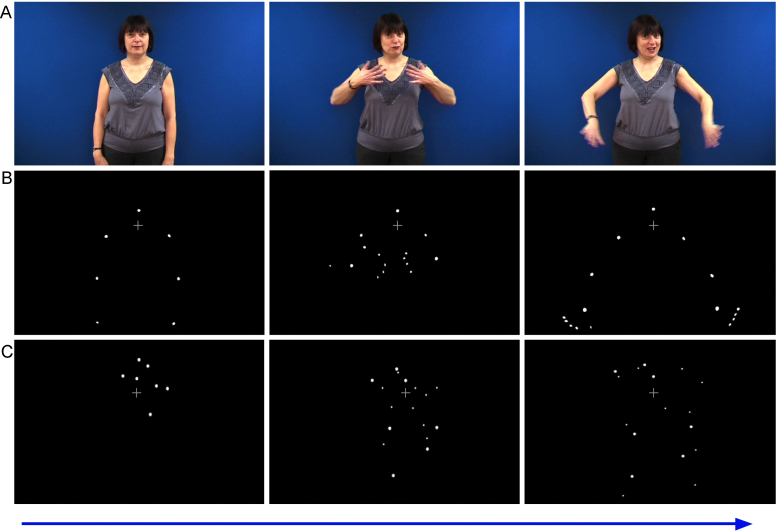
Still images taken from video showing: (A) natural SL – one of the target signers producing the BSL sign DRESS (shown for illustration purposes; not presented in the experiment), (B) the same signer producing DRESS in point-light form and (C) an example of the motion baseline.

The stimuli for this study were recorded from several different signers who produced a number of different signs that were then edited and shown as point-light displays (point-light SL). The baseline (motion control) condition reported here was a dynamic display which used the same spatial parameters as the points in the point-light display when the signer was at rest, but reconfigured to generate a figure in which the velocities of the trajectories of the points were similar to those in the biological motion display (see Fig. 1 and below for details). An additional baseline of the same number of static dots in an asymmetrically arranged configuration that could not be construed as a meaningful form was included, but activations in relation to this low-level baseline are not reported here.

The experimental stimuli comprised 10 unconnected BSL lexical items: GLOVES, FRIEND, BIRTHDAY, DRESS, BOOK, WORK, NIGHT, SHOP, NAVY, and FENCE. BSL stimuli were chosen so that their meanings were easily identifiable in point-light displays. This was determined by pretesting a group of deaf signers who were not scanned. All signs were bimanual, symmetrical and produced in neutral signing space. That is, no signs could be identified on the basis of initial hand location and/or early movement. In addition, all signs used similar handshapes; typically ‘flat’ and open, so that every finger was in full view in order to maximize visibility of the stimuli.

Each sign was produced by 10 signing models (3 deaf, 7 hearing). Eight signers were unfamiliar to participants. Two deaf signers (one male, one female) were well known in Deaf^2^ community. They frequently appeared on television programs for deaf people, and were chosen as target identities for the person identification condition in order to optimize signer recognition performance. In each of the two stimulus list presentations (see below), only one familiar model was seen, and the unfamiliar signers in each list were of a different gender to the target. All models either used BSL as their first language or had obtained a minimum BSL level 2 qualification, indicating reasonable BSL fluency. Between each sign, the model's hands came to rest at his/her side.

### 2.3 Experimental design and task

From an overall list comprising all the signers and the signs they made, two experimental stimulus lists were selected. This was in order that every participant saw a different list for the person identification (PI) and the sign identification (SI) condition, thus minimizing any effects due to priming from a previous item. List and order of experiments were counterbalanced. Within each list, the signers (one familiar, four unfamiliar) appeared ten times, each performing five signs. Hence, within a list, each sign and signer was shown ten times.

The person and sign identification tasks were performed in separate runs and counterbalanced across participants. For display within the scanner, 5 blocks of the experimental task (either sign or person identification) alternated with five blocks of the movement-control condition. Each of these blocks comprised ten items and lasted 33 s. In addition, ten blocks of a low-level baseline condition comprising an arrangement of still dots (duration 15 s), were inserted between every experimental and movement control condition. The total duration of each run was 8 min.

### 2.4 Experimental task

In the PI task, volunteers were instructed to press a button when they saw the target signer. Participants were trained to recognize the target signer prior to exposure in the scanner and were again reminded of the target's appearance in the scanner, immediately before the PI run. Training included inspection of natural video clips of the target signer producing lists of signs in both natural and point-light displays. In addition, participants were given a brief practice for the PI task in which they received feedback on their performance. The practice consisted of three blocks of items. As with the actual experiment, each block was composed of ten items (5 signs, each produced by 5 signers (one target, four non-targets)). Participants pressed a button when they saw the target signer. Feedback during training consisted of a green ‘✓’ or a red ‘X’ presented above the video for items that were correctly and incorrectly identified as targets, respectively. At no point were participants given information on how to identify the target signer. None of the signs used in the pre-scan training and practice were used in the scan experiment. In the SI task volunteers were directed to respond by pressing a button when they saw a target sign, which was the BSL sign ‘BOOK’ for half the participants and ‘FENCE’ for the other half of participants, depending on the list presented.

### 2.5 Creating point-light stimuli from natural displays

Point-light stimuli were obtained by an image-based method. Models were filmed wearing dark clothing with seven large white dots placed on their nose, shoulders, elbows and back of wrists. Ten smaller dots were attached to the fingernails of each hand. The footage was then edited^3^ to create video clips of individual signs (approximately 3 s each) and a ‘threshold’ filter was applied to each clip. This created a high contrast version of the stimuli by desaturating hue information, and converting all pixels lighter than the threshold to white and all pixels darker than the threshold to black.

The control stimulus comprised five video clips of moving dots matched in number, shape, color, spatial and general movement trajectory (expanding and contracting) to the signs produced by models in the experimental condition. These clips were created in Motion software application by drawing small circular shapes, resembling white dots on the models, and applying motion paths to them. These paths were specified to animate the outward motion of the shapes within a spatial region similar to the space used by signers to produce signs (sign-space). The shapes were arrayed around one central dot, which remained still throughout the video clip and which matched the approximate position of a similarly static dot on the nose of the models. Thus, while the precise motion trajectories may have differed between the stimuli in this baseline and the experimental conditions, this baseline was designed to control, to some degree, for non-biological movement and placement of the dots in the experimental stimuli. Illustrative still shots from the natural, point-light and high-level motion control sequences are shown in Fig. 1.

The low-level baseline condition (not used in the analysis reported here) comprised a video clip of a still image of white dots and small circular shapes. The central dot was at the approximate position of the nose of the model in experimental condition. The spread, shape, contrast and number of circles in the still image corresponded to the dots seen in experimental condition, and the display duration of this image was 15 s.

Throughout the experiment, a fixation cross was superimposed at the top third of the midline of the video. For the experimental stimuli, this encouraged fixation at the approximate location of the signer's chin – the location typically used by native signers viewing natural sign (Muir & Richardson, 2005).

The task for participants in each of these baseline conditions was to press a button when the gray fixation cross turned red. To maintain vigilance, targets in experimental and motion control conditions occurred at a rate of 2 per block at an unpredictable position. Targets in the shorter still-image control condition appeared at a rate of 1 per block. All participants practiced the tasks outside the scanner.

All stimuli were projected onto a screen located at the base of the scanner table via a Sanyo XU40 LCD projector and then projected to a mirror angled above the participant's head in the scanner.

### 2.6 Imaging parameters

Gradient echoplanar MRI data were acquired with a 1.5-T General Electric Signa Excite (Milwaukee, WI, USA) with TwinSpeed gradients and fitted with an 8-channel quadrature head coil. Three hundred T2*-weighted images depicting BOLD contrast were acquired at each of the 40 near-axial 3-mm thick planes parallel to the intercommissural (AC-PC) line (0.3 mm interslice gap; TR = 3 s, TE = 40 ms, flip angle = 90°). This field of view for the fMRI runs was 240 mm, and the matrix size was 64 × 64, with a resultant in-plane voxel size of 3.75 mm. High-resolution EPI scans were acquired to facilitate registration of individual fMRI datasets to Talairach space (Talairach & Tournoux, 1988, chapter). These comprised 40 near-axial 3-mm slices (0.3-mm gap), which were acquired parallel to the AC-PC line. The field of view for these scans was matched to that of the fMRI scans, but the matrix size was increased to 128 × 128, resulting in an in-plane voxel size of 1.875 mm. Other scan parameters (TR = 3 s, TE = 40 ms, flip angle = 90°) were, where possible, matched to those of the main EPI run, resulting in similar image contrast.

### 2.7 Data analysis

The fMRI data were first corrected for motion artifact then smoothed using a Gaussian filter (FWHM 7.2 mm) to improve the signal to noise ratio. Low frequency trends were removed by a wavelet-based procedure in which the time series at each voxel was first transformed into the wavelet domain and the wavelet coefficients of the three levels corresponding to the lowest temporal frequencies of the data were set to zero. The wavelet transform was then inverted to give the detrended time-series. The least-squares fit was computed between the observed time series at each voxel and the convolutions of two gamma variate functions (peak responses at 4 and 8 s) with the experimental design (Friston, Josephs, Rees, & Turner, 1998). The best fit between the weighted sum of these convolutions and the time series at each voxel was computed using the constrained BOLD effect model suggested by Friman, Borga, Lundberg, and Knutsson (2003). Following computation of the model fit, a goodness of fit statistic was derived by calculating the ratio between the sum of squares due to the model fit and the residual sum of squares (SSQ ratio) at each voxel. The data were permuted by the wavelet-based method described by Bullmore et al. (2001) with the exception that, prior to permutation, any wavelet coefficients exceeding the calculated threshold were removed and replaced by the threshold value (Donoho & Johnstone, 1994). This step reduced the likelihood of refitting large, experimentally unrelated components of the signal following permutation. Significant values of the SSQ were identified by comparing this statistic with the null distribution, determined by repeating the fitting procedure 20 times at each voxel. This procedure preserves the noise characteristics of the time-series during the permutation process and provides good control of Type I error rates. The voxel-wise SSQ ratios were calculated for each subject from the observed data and, following time series permutation, were transformed into standard space (Talairach & Tournoux, 1988, chapter) as described previously (Brammer et al., 1997; Bullmore et al., 1996). The Talairach transformation stage was performed in two parts. First, the fMRI data were transformed to high-resolution T2*-weighted image of each participant's own brain using a rigid body transformation. Second, an affine transformation to the Talairach template was computed. The cost function for both transformations was the maximization of the correlation between the images. Voxel size in Talairach space was 3.75 mm × 3.75 mm × 3.75 mm.

### 2.8 ANOVA: comparison of PI and SI conditions

In contrast to good (ceiling) scores on the SI task, behavioural performance on the PI task varied across participants (see below for behavioural results). Therefore, we regressed out the d-prime scores in the PI condition prior to performing analysis of variance (ANOVA). ANOVA comparing differences between experimental conditions was calculated by fitting the data at each voxel which all subjects had non-zero data using the following linear model: *Y* = *a* + *bX* + *e*, where *Y* is the vector of BOLD effect sizes for each individual, *X* is the contrast matrix for the particular inter condition contrasts required, *a* is the mean effect across all individuals in the two conditions, *b* is the computed condition difference and *e* is a vector of residual errors. The model is fitted by minimizing the sum of absolute deviations rather than the sums of squares to reduce outlier effects. The null distribution of *b* is computed by permuting data between conditions (assuming the null hypothesis of no effect of experimental condition) and refitting the above model. This permutation method thus gives an exact test (for this set of data) of the probability of the value of *b* in the unpermuted data under the null hypothesis. The permutation process permits estimation of the distribution of *b* under the null hypothesis of no mean difference. Identification of significantly activated clusters was performed by using the cluster-wise false positive threshold that yielded an expected false positive rate of <1 cluster per brain (Bullmore et al., 1999).

### 2.9 Group analysis: each experimental condition versus moving baseline

Identification of active 3-D clusters was performed by first thresholding the median voxel-level SSQ ratio maps at the false positive probability of 0.05. Contiguously activated voxels were assembled into 3-D connected clusters and the sum of the SSQ ratios (statistical cluster mass) determined for each cluster. This procedure was repeated for the median SSQ ratio maps obtained from the wavelet-permuted data to compute the null distribution of statistical cluster masses under the null hypothesis. The cluster-wise false positive threshold was then set using this distribution to give an expected false positive rate of <1 cluster per brain (Bullmore et al., 1999).

### 2.10 Correlations

We calculated the Pearson product-moment correlation coefficient between observed d-prime measures on the PI task and BOLD effect data and then computed the null distribution of correlation coefficients by permuting the BOLD data 100 times per voxel and then combining the data over all voxels. Median voxel-level maps were computed at the false probability of 0.05 and cluster-level maps, where *r* was significant, such that the expected false positive rate was <1 cluster per brain.

## 3 Results

### 3.1 Behavioural results

D-prime scores were used to measure behavioural performance responses (mean percent correct: PI = 79.17 (S.D. = 15.05), SI = 95.83 (S.D. = 9)). Despite several methodological refinements designed to make the task of identifying the signer relatively easy, performance in the PI condition was less accurate than in the SI condition (mean d-prime: PI = 3.57 (S.D. = 2.58), SI = 10.08 (S.D. = 2.08), paired samples *t*(11) = −7.83, *p* < 0.001). Nevertheless, d-prime values show identification of signer to be better than chance.

### 3.2 fMRI results

**Fig. 2:**
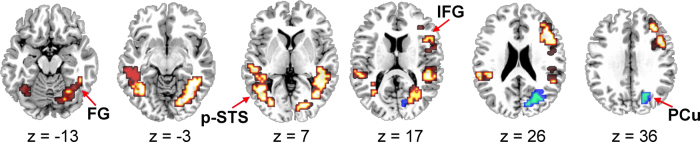
Regions showing significant activation: person identification (PI) greater than sign identification (SI) are in red/yellow, SI greater than PI are in blue/green; six axial sections, are displayed; the left hemisphere is displayed on the left; regions labeled: fusiform gyrus (FG), posterior superior temporal sulcus (STS-p), inferior frontal gyrus (IFG) and precuneus (PCu); voxelwise *p* value = 0.05, cluster-wise *p*-value = 0.005. (For interpretation of the references to color in this figure legend, the reader is referred to the web version of the article.)

(1) Task effects: person identification (PI) versus sign identification (SI)
  Having covaried for PI behavioural scores (see Section 2), ANOVA was used to compare the two experimental conditions (>the motion baseline). Clusters showing greater activation for PI than SI were focused in the inferior temporal gyri of each hemisphere (BA 37/19) and included the fusiform gyri (BA 37) and middle and posterior portions of the middle temporal gyri (BAs 37 and 19), and the superior temporal gyri (BAs 22 and 42). These clusters also extended superiorly into the supramarginal gyri (BA 40) and posteriorly into the middle occipital gyri. The right hemisphere cluster also extended into the lingual gyrus (BA 19) and cuneus (BA 17/18) whereas the left hemisphere cluster extended into the lateral portion of the transverse temporal gyrus (BA 41). An additional cluster was focused in the right middle frontal gyrus (BA 9). This extended dorsally to BA 8 and precentral gyrus (BA 6) and ventrally to DLPFC (BA 46) and the inferior frontal gyrus (BAs 44 and 45) (Fig. 2 and Table 1).
  In contrast, greater activation for SI than PI was found in only one cluster and was more limited. This cluster was focused in the right superior occipital gyrus (BA 19). It extended to the inferior parietal lobule (BA 40), into the intraparietal sulcus (BA 40/7) and medially to the precuneus (BAs 19, 7) and cuneus (BA 18).
(2) Sign identification (SI) > motion control; Person identification (PI) > motion control
  Compared to the moving baseline, the PI and SI conditions elicited a similar pattern of activation in both hemispheres, including large clusters focused in the left middle occipital gyrus (BA 19) and the right middle occipital/inferior temporal gyri (BA 19/37). These clusters extended inferiorly to include the fusiform gyrus (BAs 37, 19) and the cerebellum, and posteriorly to the medial occipital cortex including the cuneus and precuneus (BAs 17, 18, 19, 7). They extended superiorly to the posterior portion of the middle temporal gyrus (BA 21), superior temporal sulcus, and superior temporal gyrus (BA 22), the inferior parietal lobule (supramarginal (BA 40) and angular (BA 39) gyri) to the intraparietal sulcus and the border of the superior parietal lobule (BA 7). For the PI comparison, the cluster in the left hemisphere extended to include the middle portion of the middle temporal gyrus (BA 21), superior temporal sulcus and superior temporal gyrus (BAs 22, 42). Additional activation was observed for both conditions, relative to baseline, in the right frontal cortex, focused at the border of the DLPFC and the middle frontal gyrus (BA 46/9). This cluster of activation extended into the inferior frontal (BAs 44, 45) and precentral (BAs 6, 4) gyri (Table 2).

### 3.3 Correlation analysis

Since behavioural performance on the PI task varied widely across participants correlational analysis was performed in order to examine the extent to which regions involved in the PI condition could be modulated by task performance. Regions displaying positive correlations between PI performance and activation in the PI condition are summarised in Table 3. As well as activation in medial structures, including the anterior cingulate gyrus, activation in several other cortical regions showed positive correlations with PI performance. These included left inferior frontal regions, as well as left angular gyrus, extending into left parietal regions, and left inferior fusiform gyrus. Activation in the left precuneus was also positively correlated with performance on the PI task.

## 4 Discussion

Deaf native signers were able to identify both target signs and signers under point-light conditions. It is possible to target a known signer from the meagre information provided by point-light displays, even when, as in this case, the manual utterance was an isolated sign, and all signs were produced in a similar location, and with similar handshapes. However, despite our best efforts, person identification (PI) was more error-prone than sign identification (SI), suggesting that the tasks were not equivalent in terms of difficulty. Moreover, we do not know which cues respondents used to perform the PI task. Gender was a possible cue, since all foils differed from targets in gender. However, although gender can be discriminated from whole-body point-light movement, the critical information appears to be in gait and hip movement (Pollick, Kay, Heim, & Stringer, 2005) rather than in torso and hands, which carried movement in this study. Ceiling performance on the SI task suggests that we chose appropriate single signs that can be easily recognized from their kinematic properties under the point-light conditions used here. Natural SL discourse involves additional face and body movements, which were not explored in the present study, and which are likely to bear on the task if identifying the signer. In analysis of the fMRI data we dealt with the discrepancy in performance on the SI and PI tasks in two ways: first, we co-varied for the individual d-prime accuracy scores on the PI task before carrying out the PI versus SI contrasts (ANOVA). Also, we were able to correlate individual performance on PI to indicate regions of activation that were related to accuracy in identification.

In large measure, the findings reported here correspond with previous studies of the cortical correlates of biological motion perception, and confirm the prediction in Section 1. In relation to a moving point-light baseline that carried no biological significance, both tasks showed extensive activation in regions that support biological movement processing. Within temporal regions, as predicted, activation along the superior temporal gyrus was extensive, extending superiorly into parietal and inferiorly in middle-temporal regions. STS-p and the inferior parietal sulcus were included in this activation, reflecting their well-established role in biological motion processing (Grèzes et al., 2001). Our results extend these conclusions to the processing of actions of the head, arms and upper body related to potentially communicative actions – in this case, SL processing. The activation observed in posterior parts of superior temporal regions, extending superiorly into inferior parietal regions, is similar to the findings of Bonda et al. (1996), when observers viewed and recognised point-light displays of transitive actions (Demb, Desmond, Wagner, & Vaidya, 1995), again extending these findings to SL processing. Activation extending superiorly into the intraparietal sulcus may reflect a functional specialization of this region for the representation of planned actions, including intentional actions (Tunik, Rice, Hamilton, & Grafton, 2007).

Activation was not, however, confined to these regions. Although the display included no visible forms (i.e. no contour or contrast-defined shapes), nevertheless there was activation in regions associated with the perception of visible forms – including not only the LOC, but also inferior temporal regions including mid-fusiform and lingual gyri (putative ‘face’ and ‘body’ areas – Downing, Wiggett, & Peelen, 2007; Kanwisher & Yovel, 2006). This also recapitulates previous findings (e.g. Grossman & Blake, 2002; Vaina et al., 2001 – and see Section 1), which report activation in functionally defined ‘face’ and ‘body’ regions of posterior superior and inferior temporal cortices when viewing whole body point-light motion.

In relation to *language* processing – that is to the processing of full-image SL displays – conclusions must be tempered by the design of the experiment which did not allow a within-participant comparison of point-light and full-image processing. For full SL processing, many previous studies suggest that activation is similar to that reported for biological motion perception – that is it encompasses inferior and superior temporal regions, extending into inferior parietal sulci. However, in contrast to biological motion processing, full-image SL processing also generates additional activation in inferior frontal regions and, generally, more extensive activation in left than in right-hemisphere regions, with foci of activation centred in perisylvian areas (see MacSweeney et al., 2008, for review). Both tasks, when contrasted with a moving baseline condition, showed activation extending into parietal regions to a greater extent than we have reported in previous studies of SL processing, possibly reflecting greater spatial processing demands for point-light than for natural sign observation. Activation within the lateral occipital complex (LOC), namely the ventral and dorsal portions of the lateral bank of the fusiform gyrus, was also observed. Since LOC is a region implicated in (viewpoint invariant) object recognition, this may reflect the contrast of a perceived coherent figure from point-lights compared with the expanding and contracting baseline display which was not seen as a coherent visual form (Haushofer, Livingstone, & Kanwisher, 2008; Pourtois, Schwartz, Spiridon, Martuzzi, & Vuilleumier, 2009). LOC activation is notgenerally reported when a SL display is compared with a similar display of communicative action that cannot be construed as SL (see e.g. MacSweeney et al., 2004).

### 4.1 A possible role for episodic knowledge in SL processing: right frontal activation

**Fig. 3:**
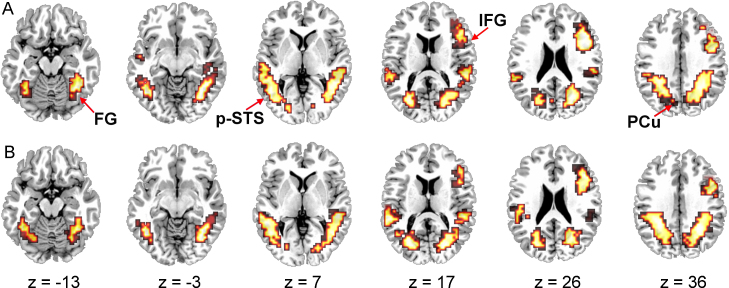
Activation for experimental conditions relative to the moving baseline task. (A) Person identification (PI) (top) and (B) sign identification (SI) (bottom); voxel-wise *p*-value = 0.05, cluster-wise *p*-value = 0.0025. Six axial sections, showing activation in temporo-occipital and frontal regions are displayed; the left hemisphere is displayed on the left; regions labeled: fusiform gyrus (FG) (Kreifelts, Ethofer, Shiozawa, Grodd, & Wildgruber, 2009), posterior superior temporal sulcus (STS-p), inferior frontal gyrus (IFG) and precuneus (PCu).

Another novel feature of the present findings compared with previous SL studies was the extent of prefrontal activation in the right, but not left, hemisphere in both PI and SI conditions (Fig. 3). Both Stevens (2004) and Von Kriegstein and Giraud (2004) report extensive right frontal activation when participants identified speaking voices as familiar or unfamiliar (compared with identifying speech content). Activation in right prefrontal regions is associated, more generally, with some aspects of episodic memory retrieval (for review, see Naghavi & Nyberg, 2005), which could be implicated in the present study as respondents try to identify instances of a specific person producing a particular utterance. We cannot rule out the possibility that, at least for respondents who performed the PI task first, there may have been some transfer of experimental ‘set’ to the sign identification task.

Frontal activation may also be related more directly to biological motion processing. In studies with patients with unilateral brain lesions (Saygin, 2007) and in neuroimaging neurologically normal groups (Saygin et al., 2004), prefrontal and superior temporal regions, bilaterally, were specifically involved in biological motion processing. Further studies could investigate the extent to which this characterises the processing of point-light SL compared with full displays, and with displays which lack biological motion (e.g. stilled SL images).

### 4.2 Person compared with sign identification – further findings

Direct contrasts between brain activation observed during PI and SI, controlled for PI performance, revealed further differences reflecting task demands. Regions activated to a greater extent by PI than SI were focused in several cortical sites. Firstly, the inferior temporal gyri, bilaterally, were activated, with activation extending into the fusiform gyri. This suggests that person identification from point-light SL preferentially involves activation of regions specialised for the processing of images of faces and bodies. Studies of voice recognition suggest that fusiform gyri are engaged only during the explicit identification of a known voice (Von Kriegstein et al., 2005). Here, the target signer was known to all participants, but the distractors were not. The extent to which these regions are sensitive to familiarity when the experimental design allows a contrast between familiar and unfamiliar signers should be examined in future studies.

Secondly, activation for PI (greater than SI) extended into the middle and posterior middle and superior temporal cortices and into the inferior parietal lobule (SMG). As well as being associated, generally, with the processing of socially relevant stimuli (see Section 1), these temporal regions are specifically associated with familiar (compared with newly learned) face recognition (Leveroni et al., 2000). Finally, activation was observed in the right inferior frontal gyrus: an areas also associated with familiar face recognition. Leveroni et al. (2000) report widespread activation of prefrontal regions, bilaterally, for familiar compared with newly learned faces. This activation included right inferior frontal regions. They attribute the activation of this somewhat right-lateralized, temporo-frontal network to the retrieval of person-specific semantic information when presented with known face images. In contrast to this pattern, identifying the sign compared with identifying the signer (SI > PI) uniquely activated a region extending from right occipital regions to the right intraparietal sulcus to the precuneus. We have not observed activation in this region when sign identification was compared (for example) with non-linguistic gesture perception (MacSweeney et al., 2004). Moreover, studies of voice recognition report an effect consistent with specialization of the right parietal regions for voice identity rather than for content processing (Stevens, 2004; Von Kriegstein & Giraud, 2004). We speculate that the right-sided occipito-parietal activation in this case may have been sensitive to the set of signed stimuli used in this study. All signs used similar handshapes, and their location was constrained. All actions were symmetrical and originated in ‘neutral’ sign space. Thus, just one SL characteristic, that of movement, was especially salient for the identification of the target. This suggests that movement, as a specific SL feature, may make distinctive use of right parietal and middle/superior occipital regions. That RH structures implicated in spatial processing can be preferentially involved in SL processing has also been shown in some previous studies (e.g. Emmorey et al., 2005). Negative findings should also be noted. SL processing did not activate perisylvian regions to a greater extent than PI processing. It is probable that observers automatically processed sign meaning when performing the PI task. Such ‘automatic’ processing of content occurs for speech processing (e.g. Shtyrov, Kujala, & Pulvermuller, 2009).

### 4.3 Other findings

The correlation analysis showed activation in several regions relating positively to behavioural performance on the PI task. The correlation of PI accuracy with anterior cingulate activation and other fronto-medial structures may well reflect effort and arousal associated with individual responses to task difficulty (Critchley, Elliott, Mathias, & Dolan, 2000). Similarly, there are suggestions that attentional modulation, involving inferior frontal and fronto-parietal circuits, may show enhanced left laterality when a task becomes difficult (see e.g. Majerus et al., 2007; Sugiura et al., 2006), and we note that left angular gyrus activation, extending into parietal regions, as well as left inferior frontal gyrus activation, is consistent with these reports. The additional focus revealed by correlation analysis in the left fusiform gyrus is consistent with the PI > SI group data. Taking the group and correlational data together, this suggests that these left-lateralized sites play an important role in the accurate processing of signer characteristics – at least under point-light conditions.

A final point to note: we did not identify activation in anterior parts of the superior temporal gyrus in this study. Nor has activation in anterior temporal regions been reliably reported in other studies of SL processing (see e.g. MacSweeney et al., 2006). Right anterior temporal activation has been reported specifically and reliably for voice identification (Belin et al.,2004; von Kriegstein, 2003, 2004; Stevens, 2004). Further studies could usefully examine the role of the temporal poles, functionally associated with (variously) semantic and syntactic processing for speech, during SL processing.

## 5 Conclusions

This study, the first to use point-light stimuli to explore the cortical correlates of signed language processing, confirmed that an extensive cortical network, involving superior temporal, inferior temporal and occipito-temporal regions, as well as some frontal and fronto-parietal regions, was activated by such displays. Many of these regions, both the perisylvian language areas and the inferior and superior temporal biological motion regions, have been shown to be active when natural images are used for SL processing, suggesting that respondents activate images of faces and bodies in action when interpreting SL from point-light displays. In this study, pointlight displays of SL also recruited right frontal regions. These regions are often associated with episodic retrieval in relation to person identification. This suggestion requires direct empirical testing contrasting both full-image and point-light displays, as well as familiar and unfamiliar signers.

The extent of activation due to signer- and sign-processing could be distinguished in this network, with more extensive activation for signer than for sign identification. However, the general pattern did not recapitulate that reported for the (auditory) processing of voice and speech identification, where more fully dissociated cortical circuits for carrier and content have been described. The extent to which these differences between speech and SL reflect modality *per se*, and/or carrier or language characteristics of the two language modes, remains to be established. For example, there are indications that *linguistic* carrier characteristics such as foreign or regional accent may be reduced or absent from signed languages, in contrast to the many idiosyncratic speaker characteristics that are be processed from voices (see Ann, 2001). This difference between SL and speech could reflect an interaction of modality (SL models are usually in full view, speech is primarily carried acoustically) and language functions in relation to carrier and content.

## Figures and Tables

**Table 1:** Regions displaying significant activation for the planned comparisons (ANOVAs) after regressing out the behavioural performance on the person identification task (d-prime accuracy scores).

	Hemisphere	Size (voxels)	*x*, *y*, *z*	BA
Person identification > sign identification				
Inferior temporal gyrus	L	289	−43, −63, 0	19/37
Inferior temporal gyrus	R	387	40, −60, 0	19/37
Middle frontal gyrus	R	157	43, 22, 26	9
Sign identification > person identification				
Superior occipital gyrus	R	150	29, −67, −23	19

Voxel-wise *p*-value = 0.05, cluster-wise *p*-value = 0.005. Foci correspond to the most activated voxel in each 3-D cluster. For each comparison, the activated regions are arranged along the *z*-axis (from inferior to superior slices).

**Table 2:** Activated regions for the experimental tasks (person identification and sign identification) compared to moving baseline.

	Hemisphere	Size (voxels)	*x*, *y*, *z*	BA
Person identification				
Middle occipital gyrus	L	794	−43, −63, −3	19
Middle occipital/inferior temporal gyrus	R	884	43, −56, −3	19/37
DLPFC/middle frontal gyrus	R	327	40, 22, 26	46/9
Sign identification				
Middle occipital gyrus	L	811	−43, −63, −3	19
Middle occipital/inferior temporal gyrus	R	833	40, −56, −7	19/37
Middle frontal gyrus	R	220	43, 26, 26	46/9

Voxel-wise *p*-value = 0.05, cluster-wise *p*-value = 0.0025. Foci correspond to the most activated voxel in each 3-D cluster. For each comparison, the activated regions are arranged along the *z*-axis (from inferior to superior slices).

**Table 3:** Regions positively associated with performance on the person identification task (d-prime).

	Hemisphere	Size (voxels)	*x*, *y*, *z*	BA
Fusiform gyrus	L	7	−33, −41, −20	20
Brain stem	L	6	−4, −26, −17	–
DLPFC/inferior frontal gyrus	R	5	43, 30, 17	46/45
Cuneus	L	9	−25, −70, 17	18
Inferior frontal gyrus	L	21	−33, 11, 23	44
Postcentral gyrus	R	8	51, −22, 33	2
Anterior cingulate gyrus	R	10	4, 22, 40	32
Angular gyrus/superior occipital gyrus	L	49	−29, −59, 36	39/19
Postcentral gyrus/inferior parietal Lobule	R	13	29, −30, 36	2/40
Precuneus	L	8	−22, −59, 50	7
Precuneus	L	6	−7, −56, 53	7

Voxel-wise *p*-value = 0.05, cluster-wise *p*-value = 0.0025. Foci correspond to the most activated voxel in each 3-D cluster. The activated regions are arranged along the *z*-axis (from inferior to superior slices).

## Footnotes

2 In accord with convention, the term ‘Deaf’ refers to users of a signed language (here BSL) who are members of the Deaf community, whereas ‘deaf’ refers to the audiological condition of hearing loss.

3 The editing software was Motion, running in Final Cut Studio, within Mac OS X.
